# Supplementary material for: The quantum-confined Stark effect in layered hybrid perovskites mediated by orientational polarizability of confined dipoles
Source: Nat Commun. 2018 Oct 11;9:4214. doi: 10.1038/s41467-018-06746-5 (PMC6181967; doi:10.1038/s41467-018-06746-5)
Supplement: Supplementary file 1 — Supplementary Information [file 41467_2018_6746_MOESM1_ESM.pdf]

Supplementary Information

The Quantum-Confined Stark Effect in Layered Hybrid Perovskites Mediated by Orientational Polarizability of Confined Dipoles

G. Walters, M. Wei, O. Voznyy, R. Quintero-Bermudez, A. Kiani, D.-M. Smilgies, R. Munir, A. Amassian, S. Hoogland, E. Sargent

correspondence to: ted.sargent@utoronto.ca

Supplementary Figures

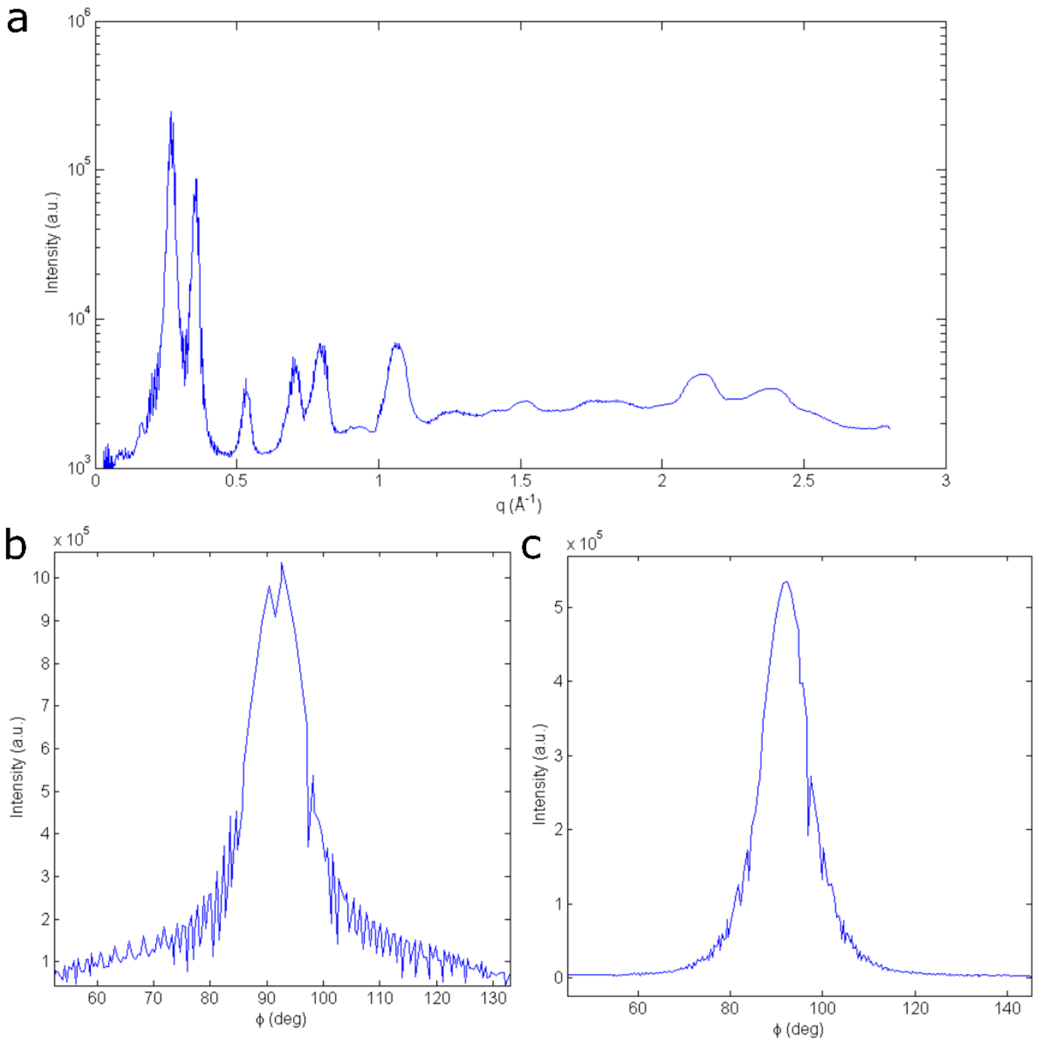

Supplementary Figure 1. GIWAXS Intensity Profiles for an *n* = 2 Perovskite Nanoplatelet Film. a, Integrated intensity as a function of scattering vector, *q*. Two intense peaks are observed at *q* values of 0.26 Å^{-1} and 0.35 Å^{-1}. These peaks correspond to scattering from the (001) plane of the well and barrier layers of *n* = 2 and 1 phases respectively. The following three peaks correspond to scattering from the harmonics of these layers. The last main peak derives from a slight ring pattern at a *q* value of 1.06 Å^{-1} and corresponds to scattering from a superposition of the (003) and the layer's in-plane {100} scattering. The relative strengths of the first two peaks indicates that the sample is primarily *n* = 2 with a smaller minority phase of *n* = 1, in agreement with the optical absorption. The FWHM of the peaks indicates that the material is highly crystalline with an average grain size of 56 nm, as found with the Debye-Scherer relation. **b,** Intensity at *q* = 0.26 Å^{-1} corresponding to scattering from the (001) plane of *n* = 2 nanoplatelets. **c,** Intensity at *q* = 0.35 Å^{-1} corresponding to scattering from the (001) plane of *n* = 1 nanoplatelets. Localization of these peaks to 90° indicates orientation of the platelets' c-axis normal to the substrate. Methylammonium and hexylammonium cations were used for GIWAXS studies.

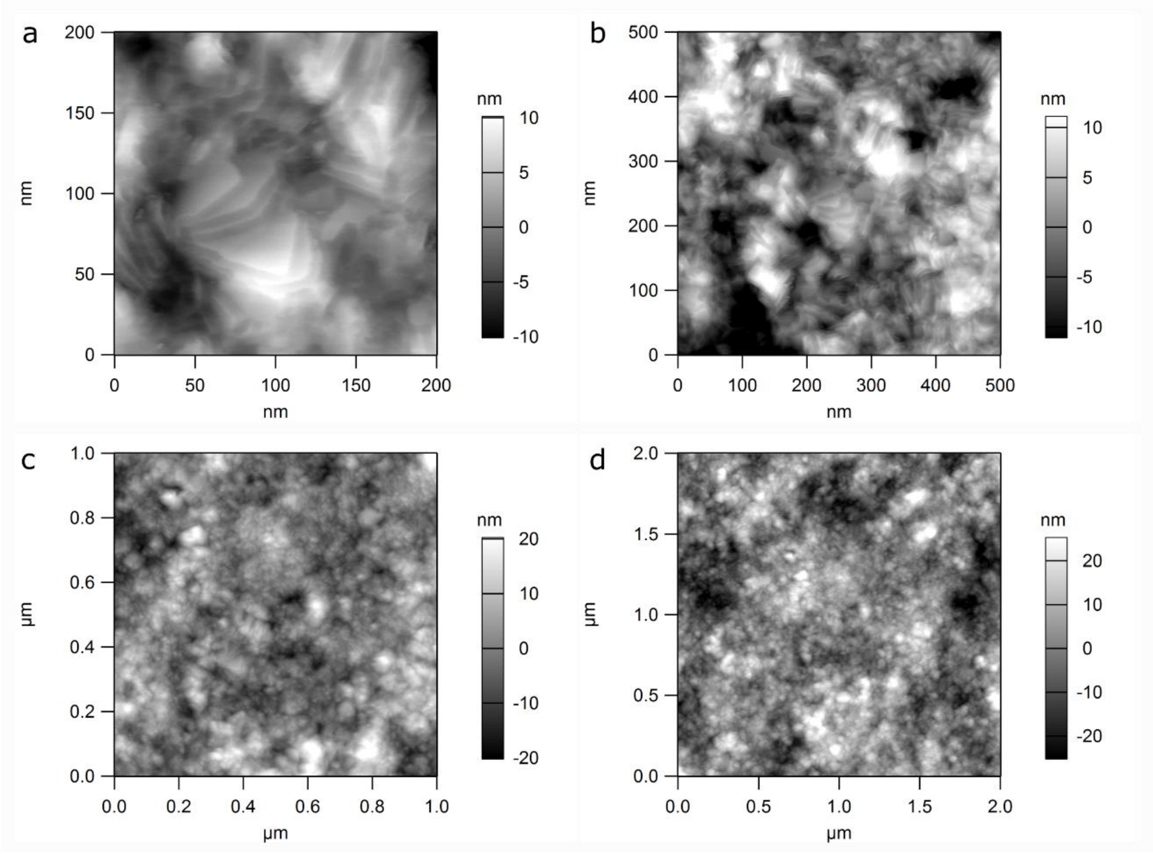

Supplementary Figure 2. AFM Topographs of *n* =3 Perovskite Nanoplatelets Thin Film. Field of views of (**a**) 200 nm, (**b**) 500 nm, (**c**) 1 µm, (**d**) 2 µm. Sample roughness was measured to be about 10 nm RMS.

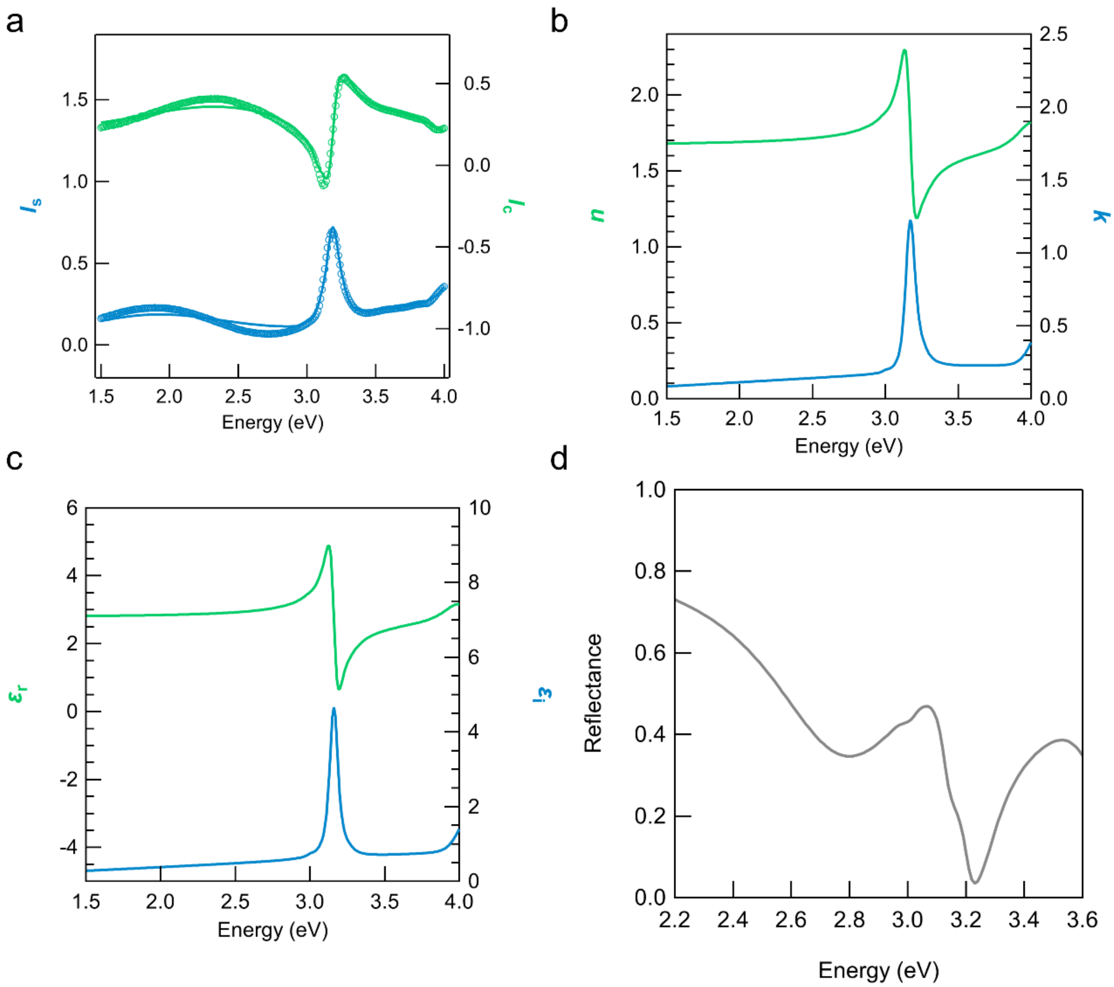

Supplementary Figure 3. Optical modelling of *n* = 1 perovskite modulator. a, Spectroscopic ellipsometry functions (circles represent data points, solid lines are model fits) for 65 degrees angle of incidence. **b,** Complex refractive index *n* and *k*. **c,** Complex dielectric function ε_r and ε_i. **d,** Simulated reflectance spectrum for the modulator device, calculated from a transfer matrix model. All plots are for perovskite nanoplatelets with a target well width of *n* = 1 with hexylammonium cations.

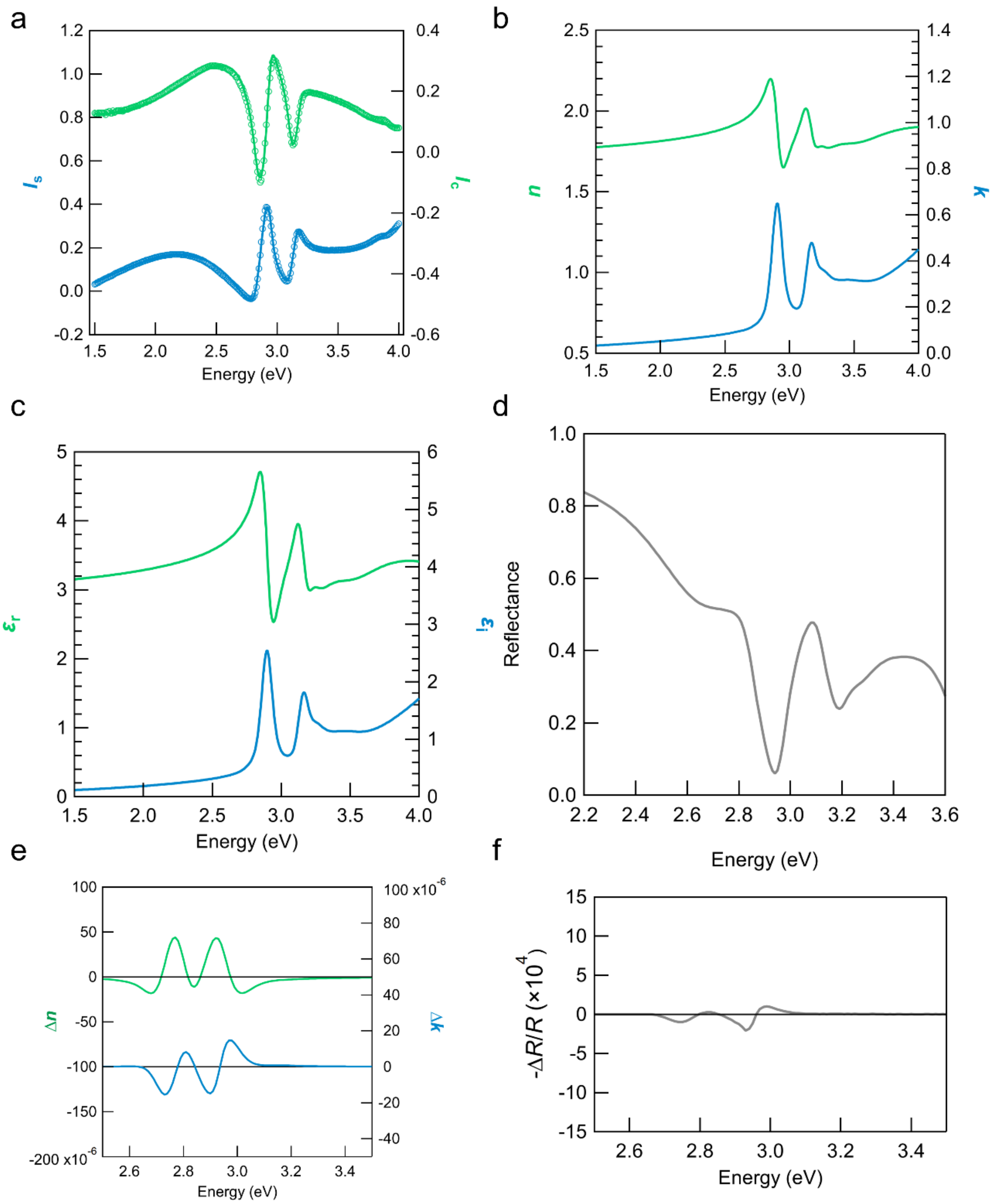

Supplementary Figure 4. Optical modelling of *n* = 2 perovskite modulator. a, Spectroscopic ellipsometry functions (circles represent data points, solid lines are model fits) for 65 degrees angle of incidence. **b,** Complex refractive index *n* and *k*. **c,** Complex dielectric function ε_r and ε_i. **d,** Simulated reflectance spectrum for the modulator device, calculated from a transfer matrix model. **e,** Calculated field-induced changes to the complex refractive index. **f,** Simulated electro-absorption spectrum calculated from transfer matrix modelling. All plots are for perovskite nanoplatelets with a target well width of *n* = 2 and methylammonium and hexylammonium cations.

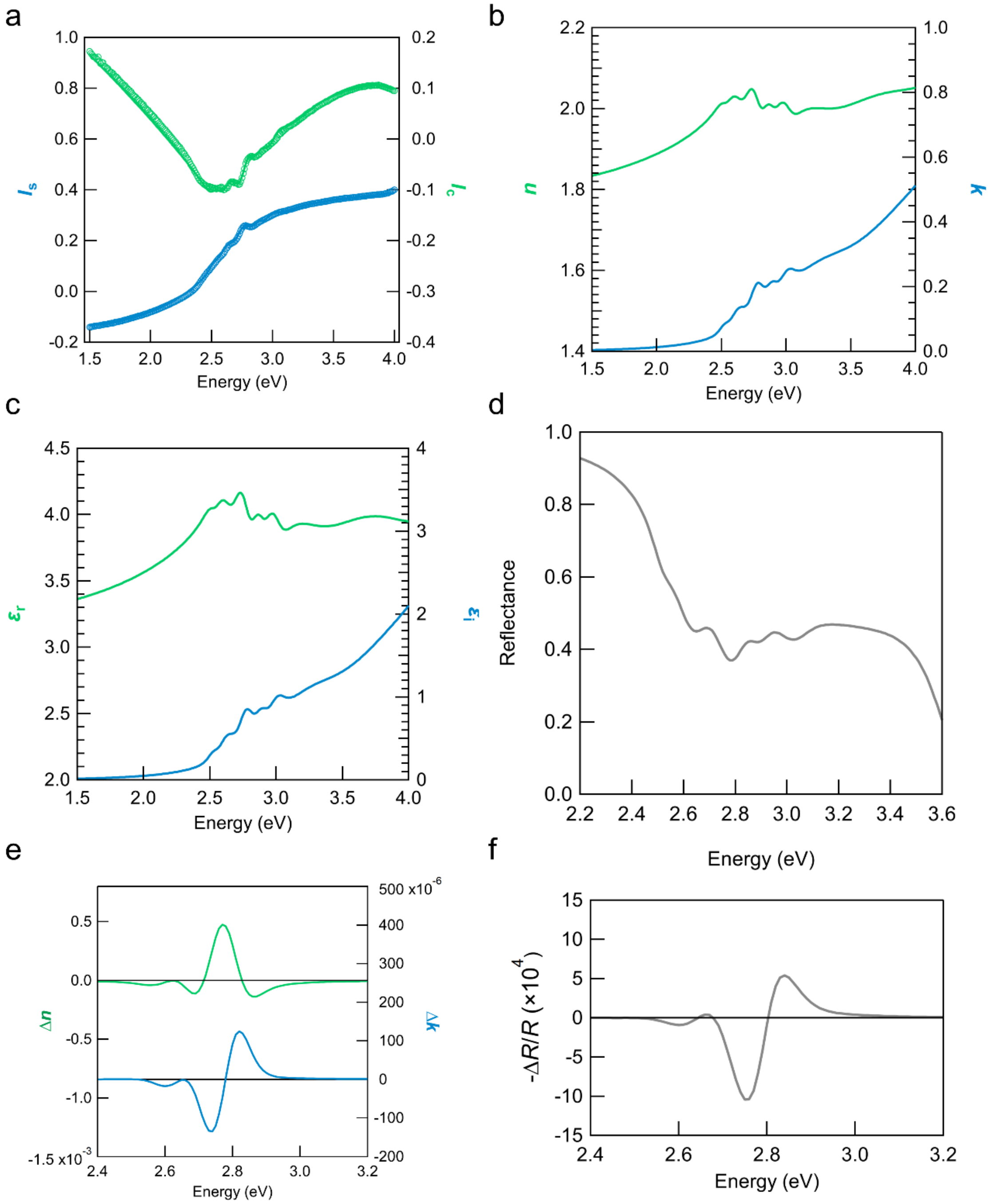

Supplementary Figure 5. Optical modelling of *n* = 3 perovskite modulator. a, Spectroscopic ellipsometry functions (circles represent data points, solid lines are model fits) for 65 degrees angle of incidence. **b,** Complex refractive index *n* and *k*. **c,** Complex dielectric function ε_r and ε_i. **d,** Simulated reflectance spectrum for the modulator device, calculated from a transfer matrix model. **e,** Calculated field-induced changes to the complex refractive index. **f,** Simulated electro-absorption spectrum calculated from transfer matrix modelling. All plots are for perovskite nanoplatelets with a target well width of *n* = 3 and methylammonium and hexylammonium cations.

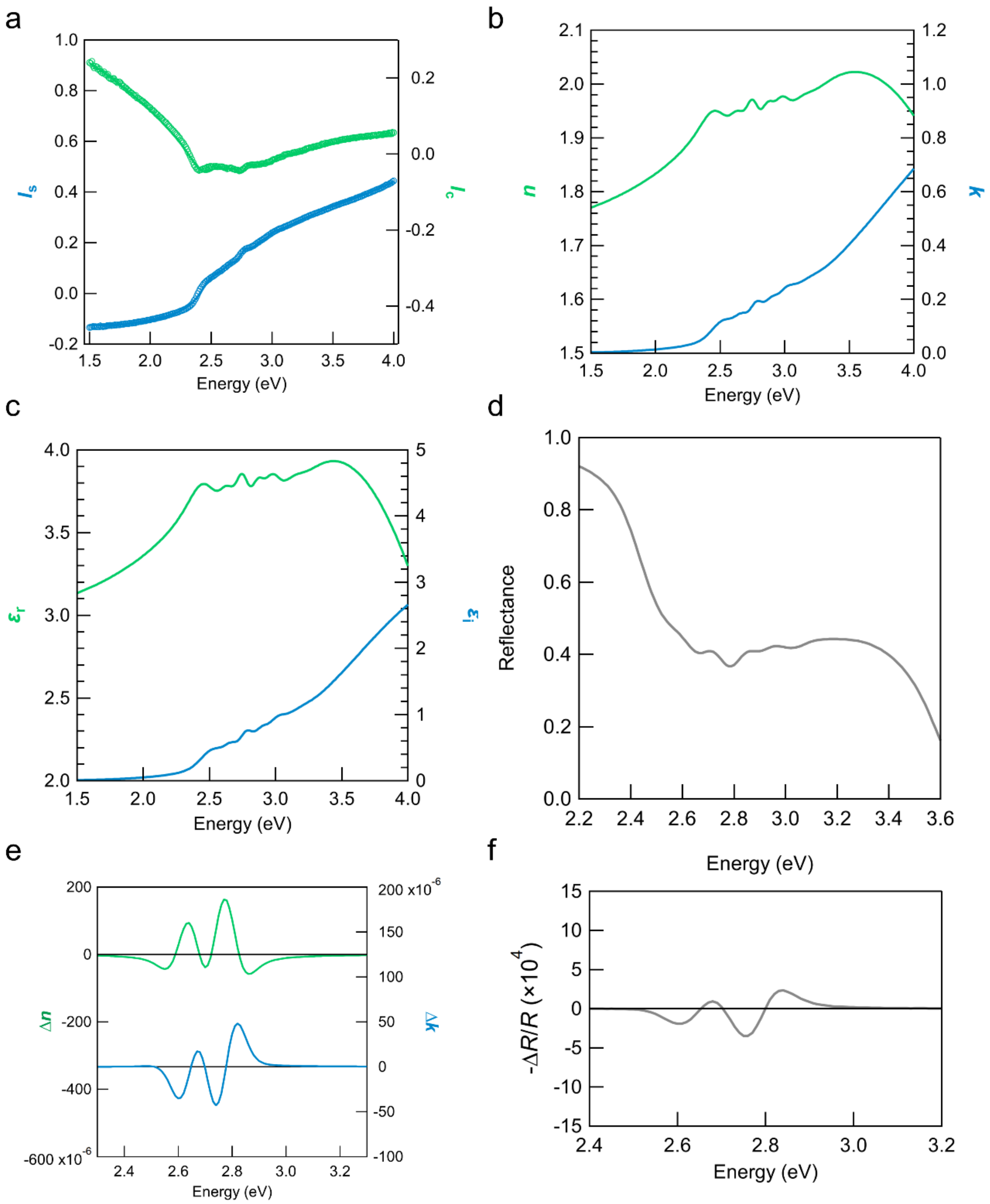

Supplementary Figure 6. Optical modelling of n = 4 perovskite modulator. a, Spectroscopic ellipsometry functions (circles represent data points, solid lines are model fits) for 65 degrees angle of incidence. **b,** Complex refractive index n and k. **c,** Complex dielectric function ε_r and ε_i. **d,** Simulated reflectance spectrum for the modulator device, calculated from a transfer matrix model. **e,** Calculated field-induced changes to the complex refractive index. **f,** Simulated electro-absorption spectrum calculated from transfer matrix modelling. All plots are for perovskite nanoplatelets with a target well width of n = 4 and methylammonium and hexylammonium cations.

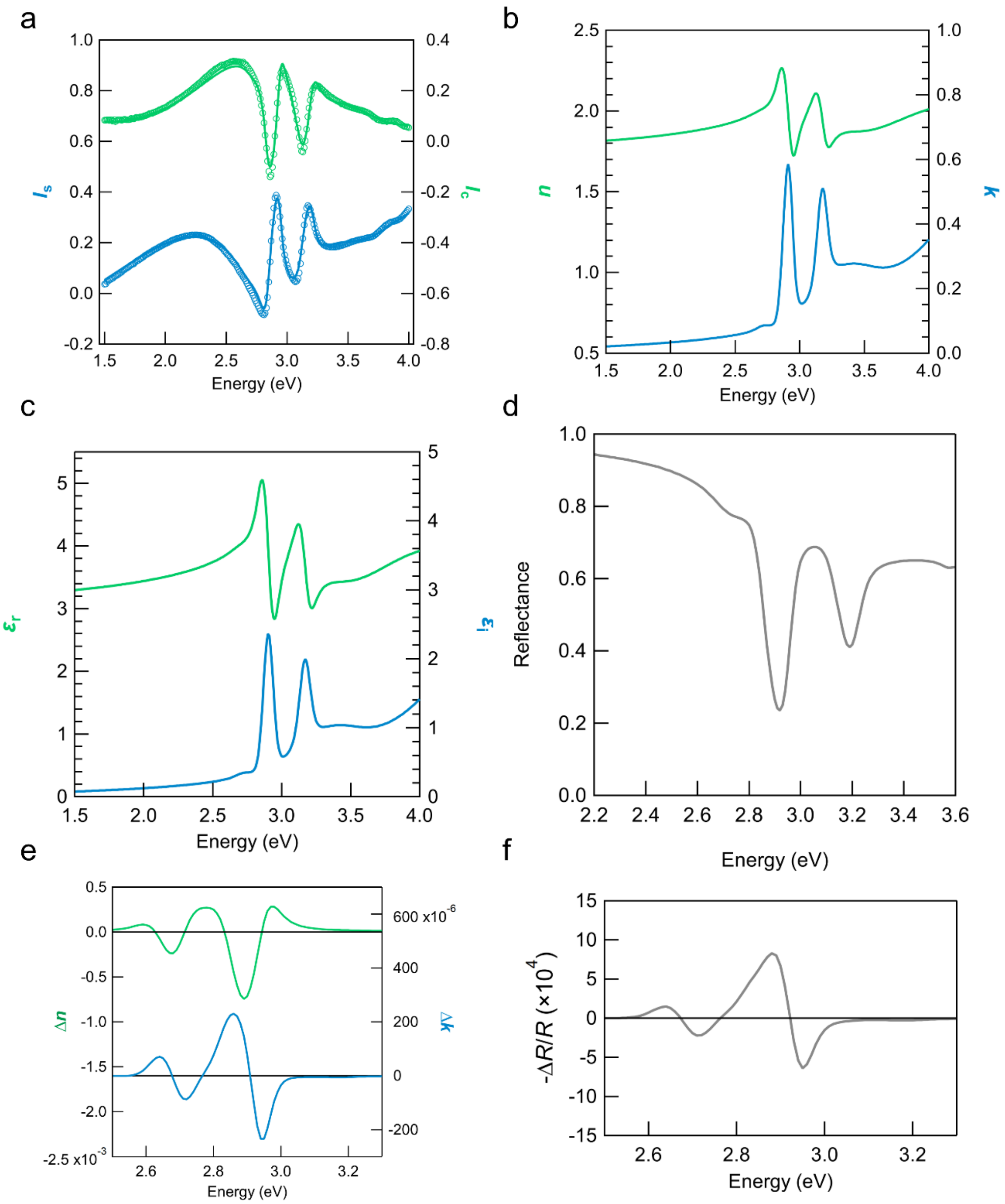

Supplementary Figure 7. Optical modelling of perovskite modulator with cesium cations. a, Spectroscopic ellipsometry functions (circles represent data points, solid lines are model fits) for 65 degrees angle of incidence. **b,** Complex refractive index *n* and *k*. **c,** Complex dielectric function ε_r and ε_i. **d,** Simulated reflectance spectrum for the modulator device, calculated from a transfer matrix model. **e,** Calculated field-induced changes to the complex refractive index. **f,** Simulated electro-absorption spectrum calculated from transfer matrix modelling. All plots are for perovskite nanoplatelets with cesium and hexylammonium cations.

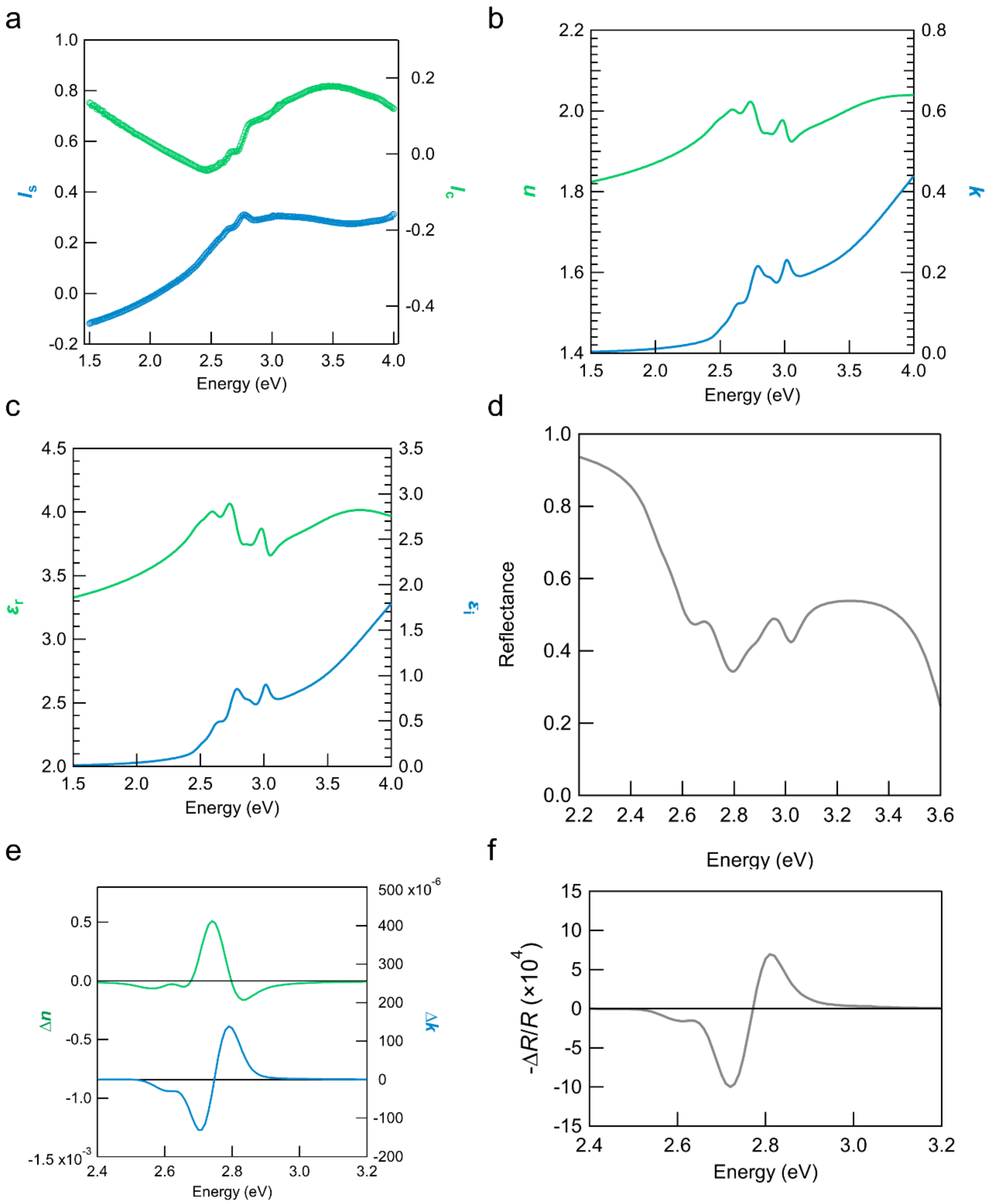

Supplementary Figure 8. Optical modelling of n = 3 octylammonium perovskite modulator. **a,** Spectroscopic ellipsometry functions (circles represent data points, solid lines are model fits) for 65 degrees angle of incidence. **b,** Complex refractive index n and k. **c,** Complex dielectric function ε_r and ε_i. **d,** Simulated reflectance spectrum for the modulator device, calculated from a transfer matrix model. **e,** Calculated field-induced changes to the complex refractive index. **f,** Simulated electro-absorption spectrum calculated from transfer matrix modelling. All plots are for perovskite nanoplatelets with a target well width of n = 3 and methylammonium and octylammonium cations.

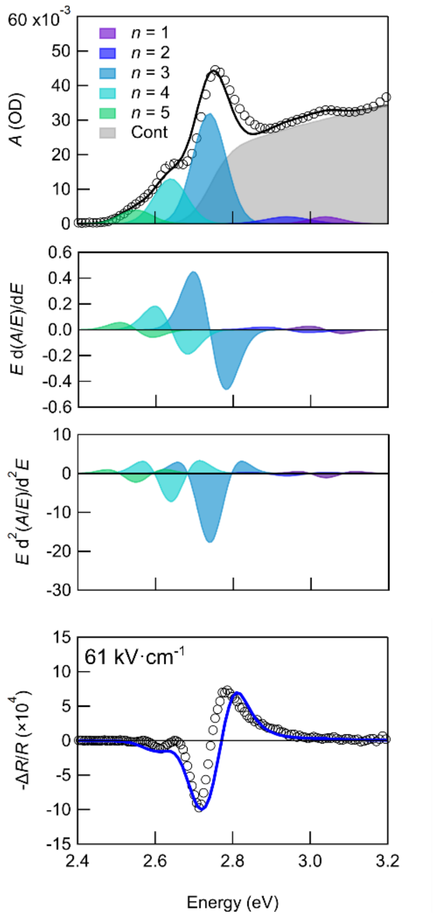

Supplementary Figure 9. EA Spectroscopy of an *n* = 3 Methylammonium Thin Film with Octylammonium Ligands. (Top to bottom) Optical absorption, first-derivative, second-derivative, and EA spectra. Open circles indicate experimental points. Solid lines indicate fits. Electric field strength indicates that applied to the nanoplatelet layers.

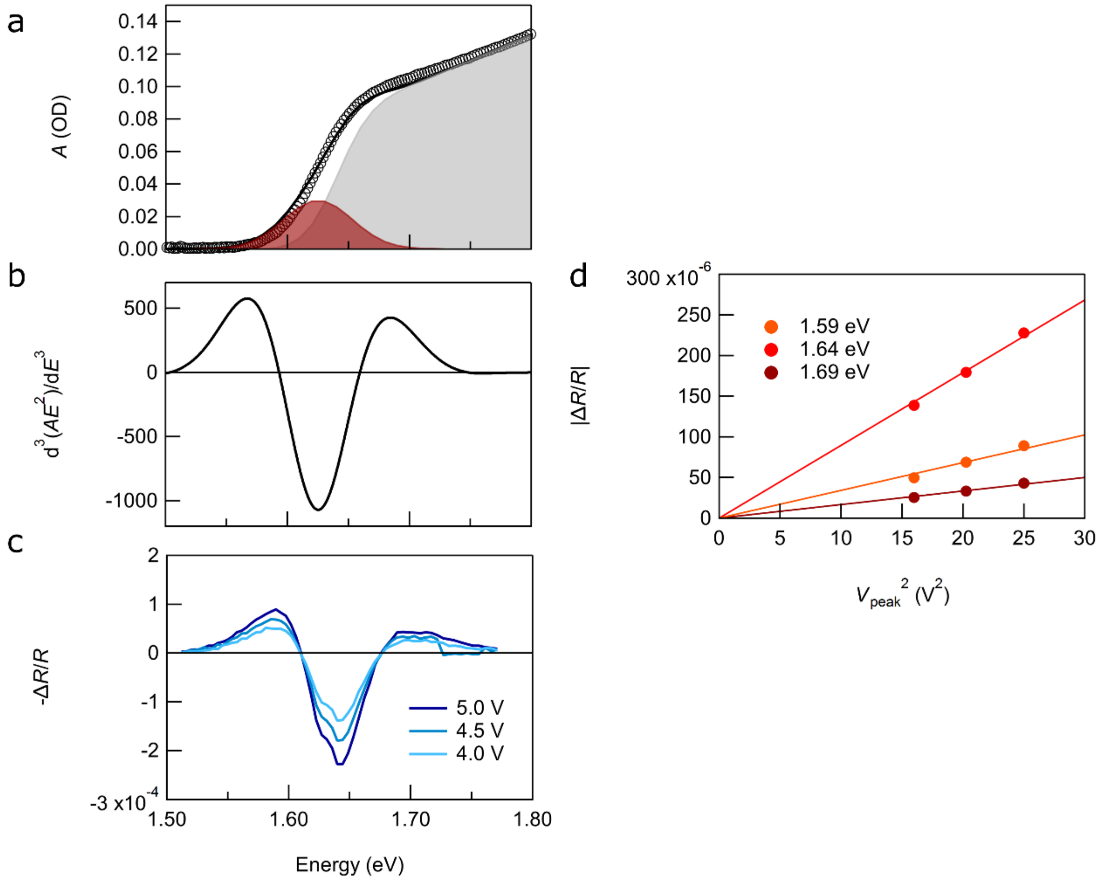

Supplementary Figure 10. EA Spectroscopy of a Thin Film of Bulk 3-D $MAPbI_3$. a, Optical absorption fit with excitonic and continuum contributions. Open circles are experimental data points. The total fit is given as the solid black trace. Unlike, for the layered hybrid perovskites, the fit here has been produced using Elliott's formula modified to account for variation from the parabolic-band regime.[1–3] **b,** Third-derivative of the optical absorption. **c,** EA spectra at various applied voltages (V_{pk}). A strong correspondence exists with the third-derivative spectrum, indicating the low-field Franz-Keldysh-Aspnes (FKA) effect[4], a result in agreement with a thorough work from Ziffer *et al.*[5]. **d,** Quadratic dependence of the EA on applied field, in agreement with the FKA effect[4].

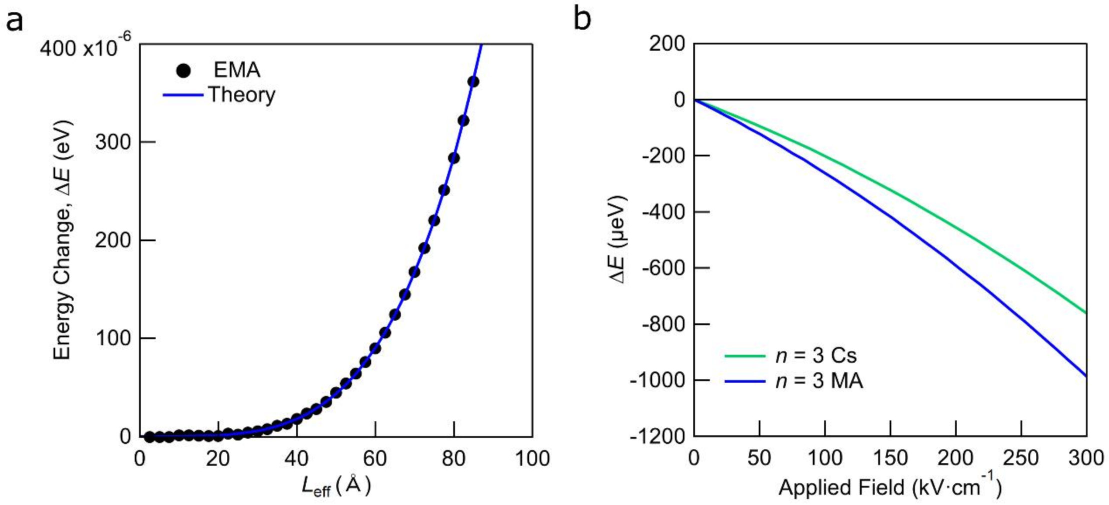

Supplementary Figure 11. EMA Calculated Stark Shifts for Layered Perovskites. a, Cumulative Stark shifts to electron and hole energy levels as a function of effective well width for an infinite well potential subjected to an electric field of 20 kV·cm^{-1}. Consistency between the calculated shifts and the theoretical relation for shifts in the quantum regime is shown. In these calculations, the effective well width is greater than the actual well width due to penetration of the wave function into the real finite well. **b**, Cumulative Stark shifts to electron and hole energy levels of n = 3 perovskites with different cations as a function of applied field. The finite well model has been used. In our electroabsorption studies, the field applied to our n = 3 perovskites is 56 kV·cm^{-1} for methylammonium films and 53 kV·cm^{-1} for cesium films. The EMA calculations indicate similar energy level shifts of ~100 µeV. This value is close to our experimentally found 300 µeV.

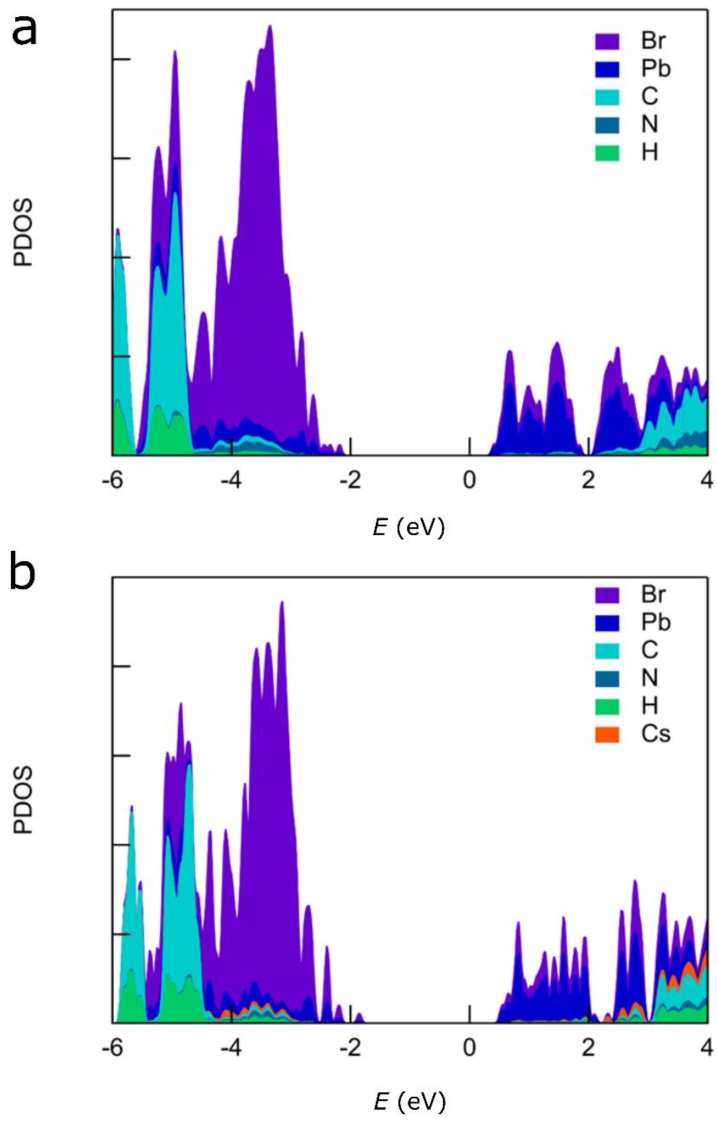

Supplementary Figure 12. Projected Density of States (PDOS) for *n* = 3 Layered Perovskites. a, PDOS for layered perovskites with methylammonium cations. **b,** PDOS for layered perovskites with cesium cations. Both plots show that the frontier orbitals are mainly composed of lead and bromine states.

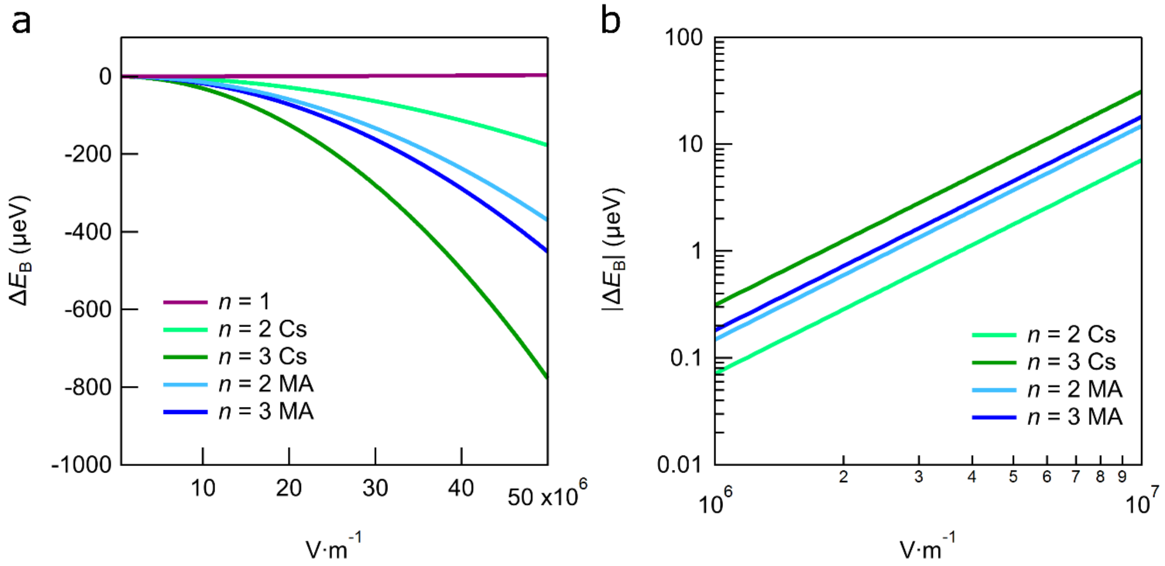

Supplementary Figure 13. DFT Calculated Change in Exciton Binding Energy for Unpolarized Layered Perovskites. a, Full scale plot of change in exciton binding energy as function of external electric field. Well widths are indicated by *n* values and cation choice is given, MA = methylammonium. **b,** Logarithmic scale plot of changes for experimentally relevant fields.

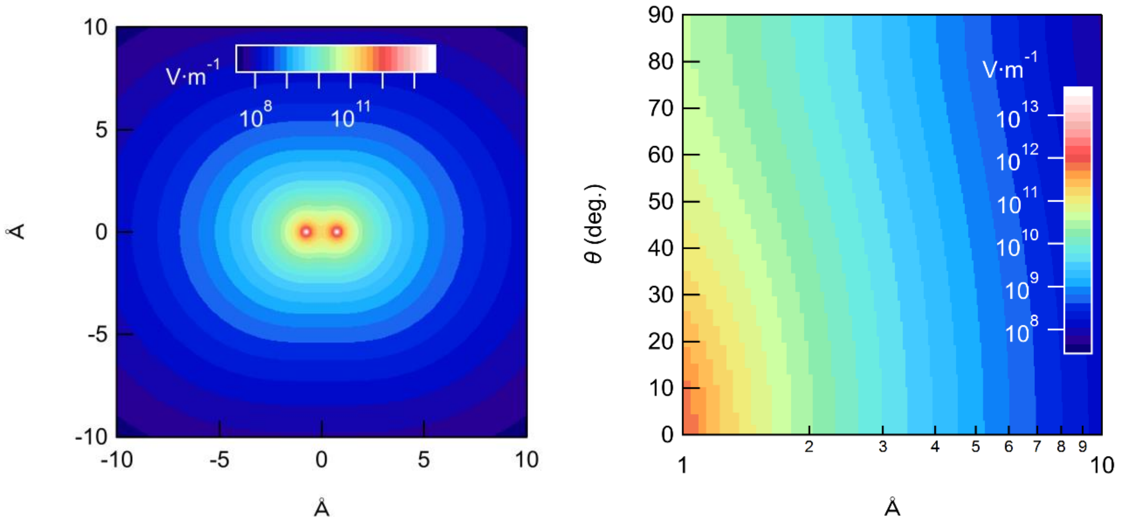

Supplementary Figure 14. Electric Field Generated by a Physical Dipole of Moment 2.3D and Length Corresponding to Methylammonium. (Left) Cartesian map of the electric field in the plane of the dipole. (Right) Angular map of the field away from the dipole axis at varying distances from the dipole center. Large differences in electric field can be found at distances that would correspond to points on the inorganic lattice of a perovskite.

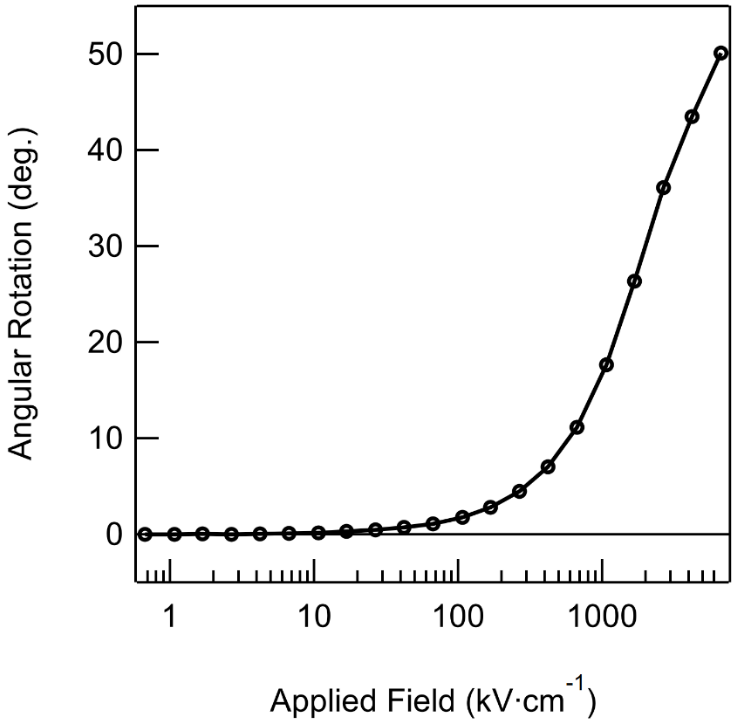

Supplementary Figure 15. Monte Carlo Simulation of Methylammonium Coupling to Electric Field. Net angular orientation away from the neutral position for a n = 3 lattice of methylammonium dipoles subjected to an applied electrostatic field.

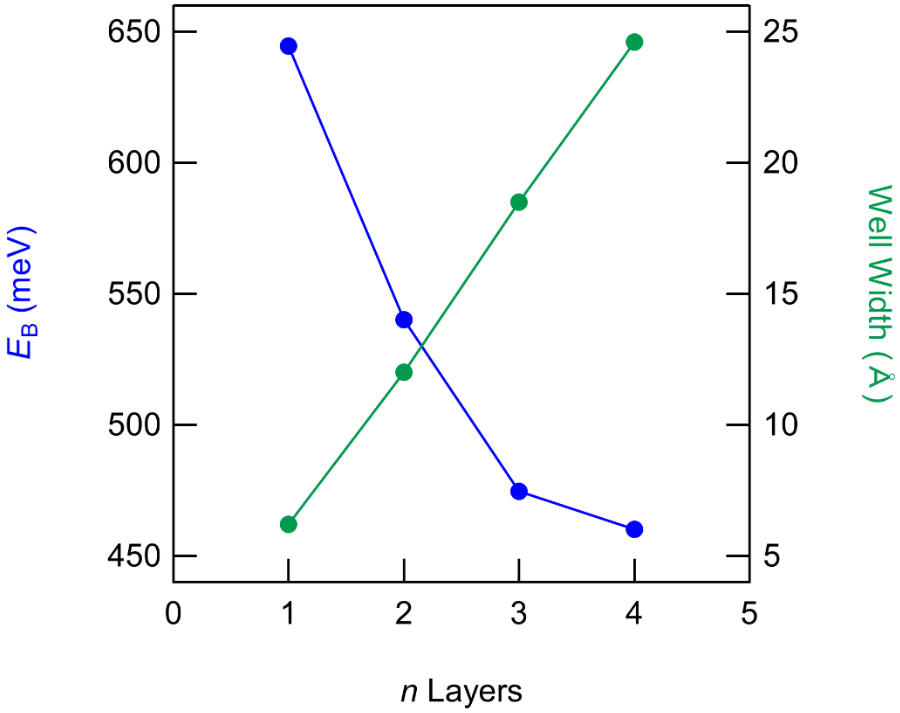

Supplementary Figure 16. Exciton Binding Energy and Well Width Dependence on the Number of Perovskite Layers. DFT calculated exciton binding energies for layered perovskites of varying well width.

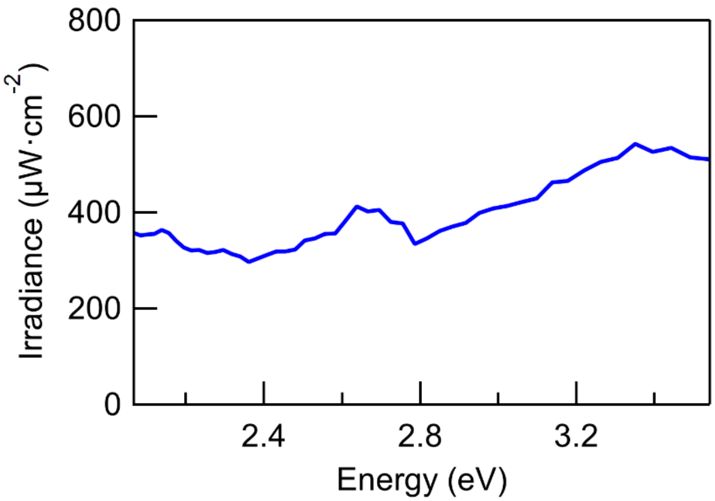

Supplementary Figure 17. Lamp Irradiance Spectrum. Irradiance spectrum for the continuous-wave xenon lamp used in our experiments. The average value for the region depicted is 380 μW·cm-2. If a 3 eV probe were to be fully extinguished by our 60 nm film, and assuming a carrier lifetime on the order of 100 ns, a maximum excitation density on the order of 10^{15} cm^{-3} is expected in our experiments.

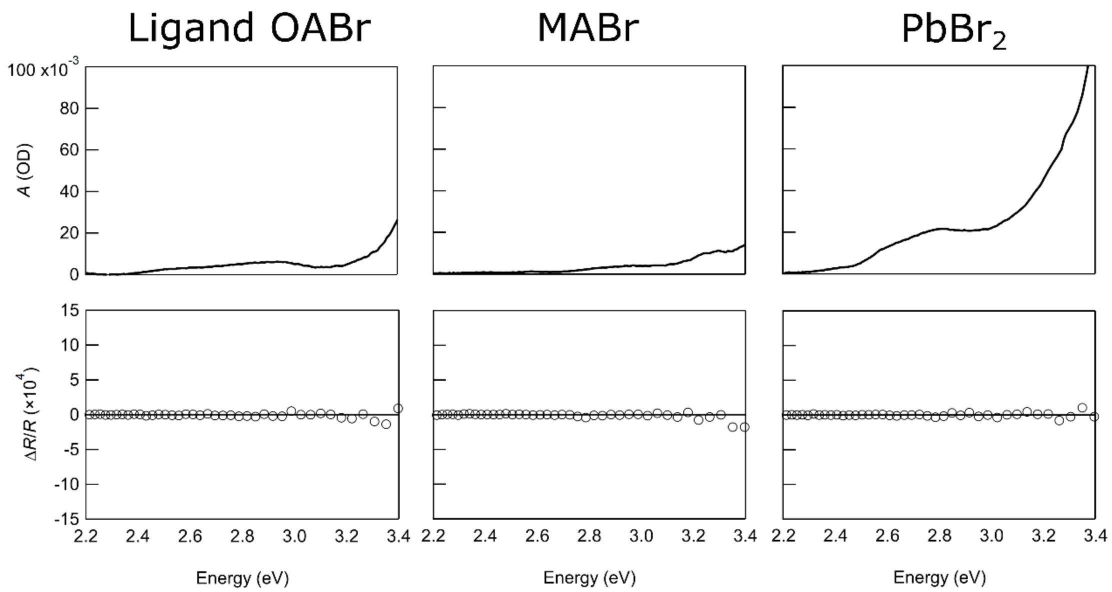

Supplementary Figure 18. Absorption and Electroabsorption for Layered Perovskite Precursors. First row, absorption spectra for octylammonium bromide ligands, methylammonium bromide, and lead bromide. Second row, electroabsorption spectra for octylammonium bromide ligands, methylammonium bromide, and lead bromide. These compounds show minimal absorption within our spectral region and no electroabsorptive signals.

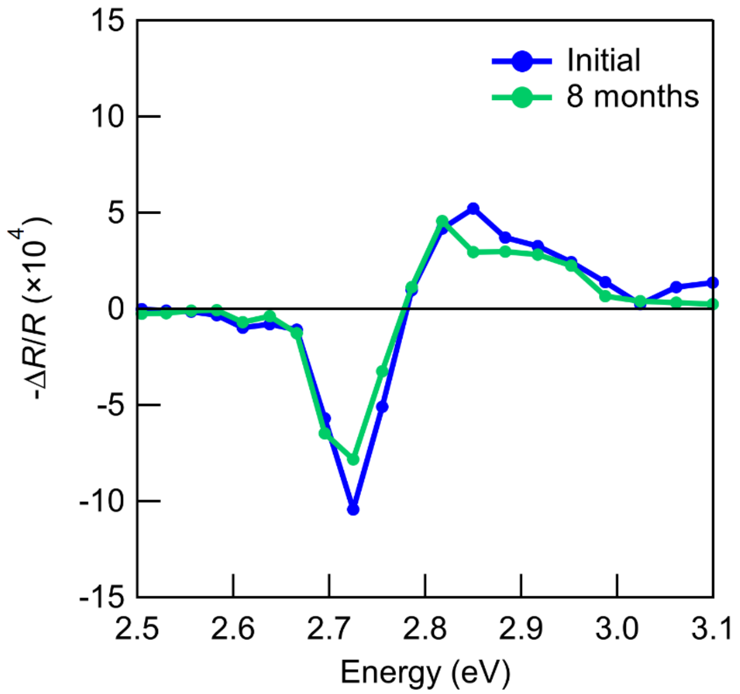

Supplementary Figure 19. Device Stability and Repeatability. Electroabsorption measurements for an n = 3 methylammonium device taken eight months apart.

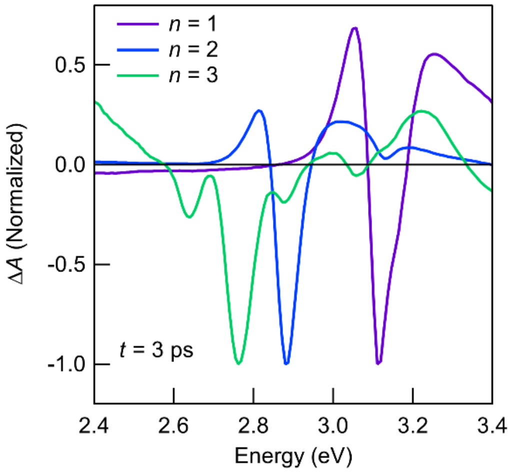

Supplementary Figure 20. Transient Absorption Traces of Perovskite Nanoplatelets. Spectra showing clearly defined peaks corresponding to the exciton resonances of perovskite wells of varying widths. Spectra were taken with a pump-probe delay of 3 ps and have been normalized to the maximum bleach point.

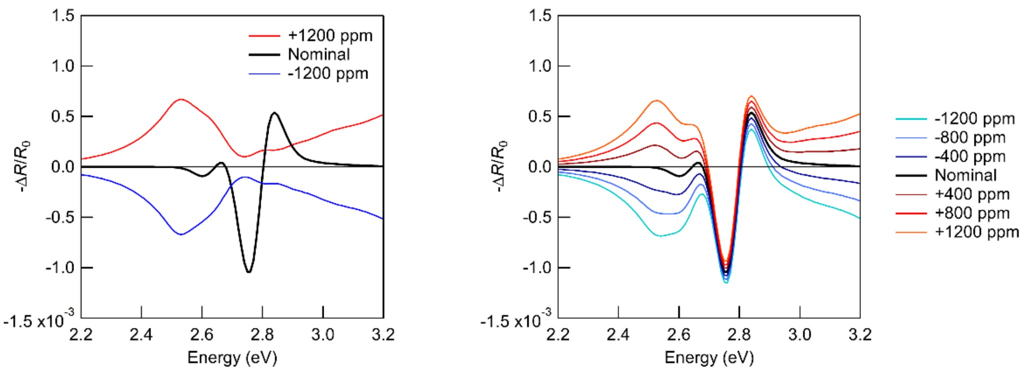

Supplementary Figure 21. Simulated Electrostriction for an *n* = 3 Methylammonium Perovskite Device. Using transfer matrix modelling, we have investigated the possibility for electrostriction coupled to the electroabsorption. Lattice expansions or contractions of up to 1200 ppm coupled with the applied electric fields have been plotted. Left plot shows the purely electrostrictive case, while the right plot shows a sensitivity analysis for electrostriction coupled with electroabsorption. The nominal trace indicates the purely electroabsorptive case, as is reported in the main text. We note that the features produced from electrostriction do not align with the experimentally observed electroabsorption data.

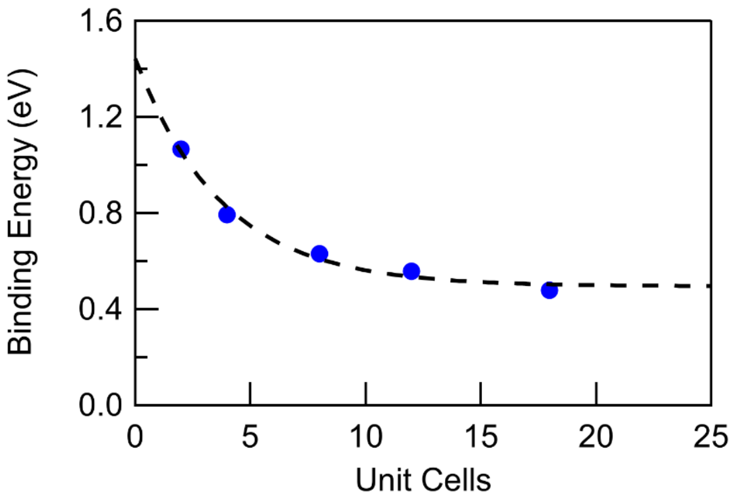

Supplementary Figure 22. DFT Calculated Exciton Binding Energy versus Supercell Size for an *n* = 3 Methylammonium Layered Perovskite. The calculations that have been presented in the main text are for an 18 unit cell ensemble.

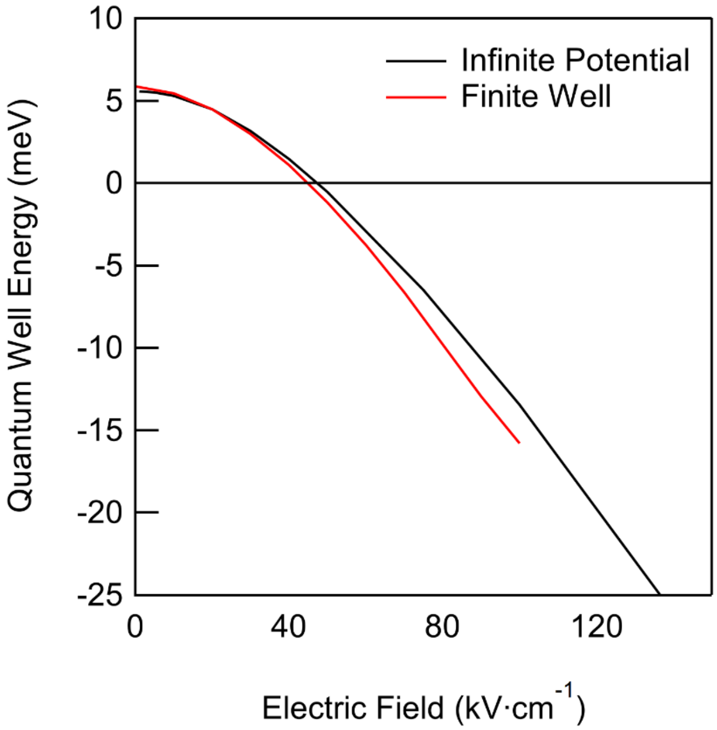

Supplementary Figure 23. EMA Calculated Energy Level Shifts for the heavy hole of GaAs/AlGaAs MQWs. We include below our reproduction of Miller *et al.*'s results[6] for the heavy-hole energy level of GaAs QWs with an 85:15 CB:VB band split. Calculations were done for both infinite and finite potential wells.

Supplementary Tables

Supplementary Table 1. Internal Electric Field. Electric fields ($kV \cdot cm^{-1}$) generated across the modulator device, within the perovskite platelets, and within the quantum wells and barriers of the platelets. Applied bias is 5 V.

	n **= 1**	***n*** **= 2, MA**	***n*** **= 3, MA**	***n*** **= 2, Cs**	***n*** **= 3, Cs**
**Nominal**	92.6	92.6	92.6	92.6	92.6
**Platelets**	76.5	64.5	56.3	61.8	53.1
**Barrier**	102.0	103.6	104.7	104.0	105.2
**Well**	13.9	16.6	16.8	10.3	10.5

Supplementary Table 2. Exciton Bohr Radii and Binding Energies. Exciton Bohr radii and binding energies for the layered perovskite materials calculated using DFT generated wavefunctions.

**Material (*n* value, MA/Cs, ligand)**	**Bohr Radius (Å)**	**Exciton Binding Energy (meV)**
n = 1, C6	14.09	644
n = 2, MA, C6	15.14	540
n = 2, Cs, C6	14.96	547
n = 3, MA, C6	16.41	476
n = 3, Cs, C6	15.68	507
n = 4, MA, C6	16.99	460
n = 3, MA, C8	16.39	478

Supplementary Table 3. Electroabsorption of Reported Thin Film Compounds and Semiconductor Devices. As a figure-of-merit, we provide the change in absorption coefficient per square of the electric field. While this list is not exhaustive, it does provide examples from a variety of thin film and conventional semiconductor materials systems. (*) indicates that the electric field was not reported and so was estimated based on reported layer thicknesses and applied voltages.

**EA Material**	**Operating Wavelength (nm)**	**Material Thickness (µm)**	**Electric Field, *F* (kV/cm)**	**$\Delta\alpha$ (cm^{-1})**	**Notes**	**$\Delta\alpha/F^2$ ($cm \cdot kV^{-2}$)**	**Ref.**
Tetracene/pentacene CT	590	0.3	50	3		1.2E-3	7
Trisdiimine ruthenium(II) CT complex with polymer matrix	450	>20	640	0.3	*T*=77K	7.3E-7	8
Bridge ruthenium complexes	1600	25	400	0.02	*T*=77K	1.2E-7	9
1D Polydiacetylene single crystals	540	0.2	24	500	T=2K	8.7E-1	10
TiOPc	700	~0.18	170	165		5.7E-3	11
CdSe NCs with polymer matrix	530	>10	11	0.04	*T*=100 K	3.3E-4	12
$(C_6H_{13}NH_3)_2PbI_4$	530	0.1	36.4	80	*T*=5K	6.0E-2	13
$(C_4H_9NH_3)_2PbI_4$	410	0.1	1010	350	*T*=5K	3.4E-4	14
$(C_6H_{13}NH_3)_2PbI_4$	530	0.2	1150	60	*T*=5K	4.5E-5	15
PbS CQDs	1635	0.7	233	11		2.0E-4	16
PCDTBT polymer fullerene	620	0.2	30*	4		3.9E-3	17
NPD/C60 mix	540	0.04	200*	20		5.0E-4	18
CdSe NPLs with a polymer matrix	470	40	120	62		4.3E-3	19
CH3NH3PbI3	750	0.2	15*	2		8.9E-3	20
CH3NH3PbI3	765	0.15	59	12		3.4E-3	5
**Perovskite Nanoplatelets (This work)**	**450**	**0.05**	**56**	**70**		**2.2E-2**	
Ge/SiGe MQW	1440	90	60	333		9.0E-2	21
Ge p-i-n	1620	45	55	384		1.3E-1	22
GeSi MQW p-i-n	1540	50	60	321		8.9E-2	23
CdZnTe/ZnTe MQW	625	500	40	120		7.5E-2	24
InGaN/GaN MQW p-i-n	400	10	400	5500		3.4E-2	25
GaN/AlGaN MQW p-i-n	270	10	500	9500		3.8E-2	25
InGaAsP	1180		193	3000		8.1E-2	26
InAsP/In MQW p-i-n	1300	300	160	4000		1.6E-2	27

Supplementary Discussion

Second-Harmonic Modulation. The use of a lock-in amplifier referenced to the second harmonic of the modulation frequency is standard practice for electroabsorption measurements as it avoids any spurious effects that may result from linear dependences on the modulation field.

Within the low-field regime electroabsorptive effects vary quadratically with field strength, whereas linear electro-optic or electromechanical effects such as the Pockels effect or piezoelectric effect vary linearly with field strength.[4,28] These effects may produce changes to the reflectance spectra of electroabsorption modulators and therefore appear as absorption changes. If these undesired effects are grouped under A_1 and the electroabsorptive effects under A_2, then,

$$\Delta A = A_1(\nu)F + A_2(\nu)F^2 \; . \qquad (1)$$

The electric field used in modulation spectroscopy can be written as,

$$F_{\mathrm{Tot}} = F_{\mathrm{App}} \sin(\omega t) \qquad (2)$$

where ω is the modulation frequency. The square of this is then,

$$F^2 = F_{\mathrm{App}}^2 \sin^2(\omega t) \qquad (3)$$

which is equivalent to,

$$F^2 = F_{\mathrm{App}}^2 \left[\frac{1-\cos(2\omega t)}{2}\right] . \qquad (4)$$

The total absorption/reflection changes, ΔA, can then be written as:

$$\Delta A = A_1(\nu)F_{\mathrm{App}} \sin(\omega t) + A_2(\nu)F_{\mathrm{App}}^2 \left[\frac{1-\cos(2\omega t)}{2}\right] \qquad (5)$$

By locking into the second harmonic of the modulation frequency, only changes related to the electroabsorptive effects will be detected.

Similarly, if the device had a built in field, F_{Dev}, the total field would be,

$$F_{\mathrm{Tot}} = F_{\mathrm{App}} \sin(\omega t) + F_{\mathrm{Dev}} \qquad (6)$$

Again considering the quadratic dependence of electroabsorptive changes on field,

$$F^2 = F_{\mathrm{App}}^2 \left[\frac{1-\cos(2\omega t)}{2}\right] + F_{\mathrm{Dev}}^2 + 2F_{\mathrm{App}}\, F_{\mathrm{Dev}} \sin(\omega t) \qquad (7)$$

only electroabsorptive changes related to the modulation bias will be detected when locking into the second harmonic.

Supplementary Methods

Transient Absorption. Femtosecond pulses were produced from a regeneratively amplified Yb/KGW laser (Light Conversion, Pharos) at a repetition rate of 5 kHz, and lowered to 1 kHz with a pulse picker. Part of this 1030 nm was sent to an optical bench (Ultrafast, Helios) where a white light continuum probe was established with a calcium fluoride crystal after passing through a delay line. The remaining portion of the fundamental was used to generate the pump at 350 nm via an optical parametric amplifier (Light Conversion, Orpheus). The pump was sent to the optical bench, chopped at 500 Hz, and lightly focused on the sample (effective beam radius 505 μm). Measurements were done in transmission mode with films deposited on glass substrates. Time delay between the pump and probe were automatically controlled with the delay line in the probe path. The probe pulse was recorded with a CCD following being dispersed with a grating spectrograph.

Supplementary References

1. Yang, Y. *et al.* Large polarization-dependent exciton optical Stark effect in lead iodide perovskites. *Nat. Commun.* **7,** 12613 (2016).
2. Saba, M. *et al.* Correlated electron–hole plasma in organometal perovskites. *Nat. Commun.* **5,** 5049 (2014).
3. Elliott, R. J. Intensity of optical absorption by excitons. *Phys. Rev.* **108,** 1384–1389 (1957).
4. Aspnes, D. E. Third-derivative modulation spectroscopy with low-field electroreflectance. *Surf. Sci.* **37,** 418–442 (1973).
5. Ziffer, M. E., Mohammed, J. C. & Ginger, D. S. Electroabsorption Spectroscopy Measurements of the Exciton Binding Energy, Electron-Hole Reduced Effective Mass, and Band Gap in the Perovskite CH3NH3PbI3. *ACS Photonics* **3,** 1060–1068 (2016).
6. Miller, D. A. B. *et al.* Band-edge electroabsorption in quantum well structures: The quantum-confined Stark effect. *Phys. Rev. Lett.* **53,** 2173–2176 (1984).
7. Sebastian, L., Weiser, G. & Bässler, H. Charge transfer transitions in solid tetracene and pentacene studied by electroabsorption. *Chem. Phys.* **61,** 125–135 (1981).
8. Oh, D. H. & Boxer, S. G. Stark effect spectra of Ru (diimine) 32+ complexes. *J. Am. Chem. Soc.* **111,** 1130–1131 (1989).
9. Oh, D. H. & Boxer, S. G. Electrochromism in the near-infrared absorption spectra of bridged ruthenium mixed-valence complexes. *J. Am. Chem. Soc.* **112,** 8161–8162 (1990).
10. Weiser, G. Stark effect of one-dimensional Wannier excitons in polydiacetylene single crystals. *Phys. Rev. B* **45,** 14076–14085 (1992).

11. Saito, T., Sisk, W., Kobayashi, T., Suzuki, S. & Iwayanagi, T. Photocarrier generation processes of phthalocyanines studied by photocurrent and electroabsorption measurements. *J. Phys. Chem.* **97,** 8026–8031 (1993).

12. Colvin, V. L., Cunningham, K. L. & Alivisatos, A. P. Electric field modulation studies of optical absorption in CdSe nanocrystals: Dipolar character of the excited state. *J. Chem. Phys.* **101,** 7122–7138 (1994).

13. Tanaka, K. *et al.* Two-dimensional Wannier excitons in a layered-perovskite-type crystal (C6H13NH3_2PbI4. *Solid State Commun* **122,** 249–252 (2002).

14. Tanaka, K. *et al.* Electronic and excitonic structures of inorganic–organic perovskite-type quantum-well crystal (C4H9NH3) 2PbBr4. *Jpn. J. Appl. Phys.* **44,** 5923–5932 (2005).

15. Tanaka, K. *et al.* Image charge effect on two-dimensional excitons in an inorganic-organic quantum-well crystal. *Phys. Rev. B* **71,** 045312 (2005).

16. Klem, E. J. D., Levina, L. & Sargent, E. H. PbS quantum dot electroabsorption modulation across the extended communications band 1200–1700nm. *Appl. Phys. Lett.* **87,** 053101 (2005).

17. Tsang, S.-W., Chen, S. & So, F. Energy Level Alignment and Sub-Bandgap Charge Generation in Polymer:Fullerene Bulk Heterojunction Solar Cells. *Adv. Mater.* **25,** 2434–2439 (2013).

18. Bernardo, B. *et al.* Delocalization and dielectric screening of charge transfer states in organic photovoltaic cells. *Nat. Commun.* **5,** 3245 (2014).

19. Achtstein, A. W. *et al.* Electroabsorption by 0D, 1D, and 2D Nanocrystals: A Comparative Study of CdSe Colloidal Quantum Dots, Nanorods, and Nanoplatelets. *ACS Nano* **8,** 7678–7686 (2014).

20. Wu, X. *et al.* Composition-Dependent Light-Induced Dipole Moment Change in Organometal Halide Perovskites. *J. Phys. Chem. C* **119,** 1253–1259 (2015).

21. Chaisakul, P. *et al.* 23 GHz Ge/SiGe multiple quantum well electro-absorption modulator. *Opt. Express* **20,** 3219–3224 (2012).

22. Feng, N.-N. *et al.* 30 GHz Ge electro-absorption modulator integrated with 3um silicon-on-insulator waveguide. *Opt. Express* **19,** 7062–7067

23. Liu, J. *et al.* Waveguide-integrated, ultralow-energy GeSi electro-absorption modulators. *Nat. Photonics* **2,** 433–437 (2008).

24. Lee, D. *et al.* Quantum well waveguide intensity modulator at visible wavelengths using CdZnTe/ZnTe quantum wells. *Appl. Phys. Lett.* **59,** 1867–1869 (1991).

25. Ozel, T., Sari, E., Nizamoglu, S. & Demir, H. V. Violet to deep-ultraviolet InGaN⁄GaN and GaN⁄AlGaN quantum structures for UV electroabsorption modulators. *J. Appl. Phys.* **102,** 113101 (2007).

26. Knüpfer, B. *et al.* Electroabsorption in InGaAsP: electro-optical modulators and bistable optical switches. *Appl. Phys. Lett.* **62,** 2072–2074 (1993).

27. Hou, H. Q., Cheng, A. N., Wieder, H. H., Chang, W. S. C. & Tu, C. W. Electroabsorption of InAsP/InP strained multiple quantum wells for 1.3 μm waveguide modulators. *Appl. Phys. Lett.* **63,** 1833–1835 (1993).

28. Kyser, D. S. & Rehn, V. Piezoelectric effects in electroreflectance. *Solid State Commun.* **8,** 1437–1441 (1970).
